# Aspartame and Human Health: A Mini-Review of Carcinogenic and Systemic Effects

**DOI:** 10.3390/jox15040114

**Published:** 2025-07-07

**Authors:** Nour El Doueihy, Joya Ghaleb, Karl Kfoury, Katy Kaleen Khouzami, Nicolas Nassif, Philippe Attieh, Hilda E. Ghadieh, Sami Azar, Amjad Kanaan, Frederic Harb

**Affiliations:** Faculty of Medicine and Medical Sciences, University of Balamand, Tripoli P.O. Box 100, Lebanon; nour.eldoueihy@std.balamand.edu.lb (N.E.D.); joya.ghaleb@std.balamand.edu.lb (J.G.); karl.kfoury@std.balamand.edu.lb (K.K.); katykaleen.khouzami@std.balamand.edu.lb (K.K.K.); nicolas.nassif@std.balamand.edu.lb (N.N.); philippe.attieh@std.balamand.edu.lb (P.A.); hilda.ghadieh@balamand.edu.lb (H.E.G.); sami.azar@balamand.edu.lb (S.A.)

**Keywords:** aspartame, carcinogenicity, oxidative stress, lipid peroxidation, formaldehyde metabolism, cancer risk, artificial sweeteners

## Abstract

Aspartame, a widely used artificial sweetener, has been at the center of ongoing debates concerning its safety, particularly its potential role in cancer development. This review provides an overview and analysis of the current research exploring the carcinogenic effects of aspartame. It examines findings from in vitro studies, in vivo experiments, and epidemiological investigations to offer a comprehensive perspective on the controversy. The results from these studies remain inconsistent—some suggest a possible association between high aspartame intake and increased cancer risk, while others fail to establish a conclusive link. Additionally, this review explores potential mechanisms by which aspartame could exert carcinogenic effects, focusing on its metabolic byproducts and their influence on cellular and molecular processes. Despite these investigations, the question of aspartame’s safety remains unresolved. Continued research is essential to clarify its role in cancer risk and to inform evidence-based dietary guidelines.

## 1. Introduction

Aspartame is one of the most widely used artificial sweeteners in the world, commonly found in a variety of food and beverage products. Aspartame became popular because of its intense sweetness at low levels, enabling it to function effectively with minimal calorie content [1]. Aspartame’s appeal stems from its advantages over saccharin, a non-nutritive artificial sweetener discovered in the late 19th century [2], as it is 200 to 300 times sweeter, contains fewer calories, and is more economical to produce [3].

Historically, saccharin was initially banned due to safety concerns, but it regained popularity during World War I when sugar shortages required alternatives. In the 1950s, cyclamate, another low-calorie sweetener, was granted GRAS (Generally Recognized as Safe) status. However, this designation was reversed when studies showed that cyclamate increased the risk of bladder cancer in laboratory rats, resulting in its ban in several countries. This history of fluctuating approval and concern laid the groundwork for the intense scrutiny surrounding aspartame following its introduction, with public perception and regulatory decisions often shaped as much by precedent and caution as by emerging scientific data [4].

Aspartame, accidentally discovered in 1965 by chemist James Schlatter, is a synthetic compound with over 70 patents covering its manufacturing process. It is composed of two amino acids, aspartic acid and phenylalanine, linked to a methyl ester. Upon ingestion, aspartame is metabolized into amino acids and methanol, which are further converted into biologically active compounds with varying effects on human health (Table 1). While the individual components of aspartame are naturally occurring in many foods, questions have been raised about the safety of their combination in the form of aspartame [4].

The controversy surrounding aspartame has existed since its discovery. Initially developed as an alternative to saccharine, which was already under debate for its safety, aspartame has been viewed as the “lesser of two evils” [6]. The controversy arises from the perceived disorganized research methods used to assess its safety, such as incomplete monkey studies that terminated prematurely and lacked proper controls and falsified glucose data in hamster trials, all leading to unsuccessful analytic conclusions and regulatory scrutiny from the Department of Health, Education, and Welfare. Regardless, the U.S. Food and Drug Administration (FDA) still approved aspartame as a food additive, and its consumption and use have continued to increase over time. Nowadays, aspartame is found in over 5000 products globally annually [10]. Its market size volume reached 770 thousand tons in 2023 [11], and its market size is projected to reach USD 476.4 million by 2029 [12]. Moreover, it remains uncertain whether the increased use of aspartame is driven by its popularity as a sugar substitute, its perceived necessity in daily diets, or the trust inspired by its FDA approval [6].

In recent years, there has been growing public concern about the potential health effects of artificial sweeteners, including aspartame. Some studies have suggested that long-term consumption of aspartame may be associated with an increased risk of various health conditions, including cancer [6]. Compelling evidence from the Ramazzini Institute demonstrates dose-related increases in malignant tumors such as lymphomas, leukemias, and renal carcinomas in rodents at exposure levels near the current ADI, with prenatal exposures showing particular sensitivity [10], while network toxicology studies reveal aspartame’s ability to bind key cancer-related proteins (AKT1, MMP9, CASP3) with high affinity, potentially disrupting cellular pathways involved in gastric carcinogenesis [13]. Thus, understanding the molecular mechanisms underlying aspartame’s potential carcinogenic effects is crucial for assessing its safety. Several studies have investigated the potential carcinogenic effects of aspartame, yielding conflicting findings. While some research, particularly in animal models, has suggested a possible association between aspartame consumption and an increased risk of cancers such as leukemia, lymphoma, and brain tumors, including a 92.3% confirmation rate of malignancy in re-evaluated rodent studies using immunohistochemical analysis [10], other studies have found no evidence to support such a link [14].

Despite these conflicting findings, the scientific evidence regarding the carcinogenicity of aspartame remains inconclusive. Regulatory agencies, including the FDA and the European Food Safety Authority (EFSA), have reviewed and concluded that aspartame is safe for human consumption within established limits [14], though these assessments have been challenged by findings showing increased hematologic malignancies at doses near current ADIs [10].

The precise molecular mechanisms underlying aspartame’s potential carcinogenic effects remain unclear. One hypothesis involves the production of formaldehyde, a known carcinogen, as a byproduct of aspartame metabolism [6]. Formaldehyde can interact with cellular components, such as DNA and proteins, potentially causing genetic mutations and cellular damage [14]. Furthermore, aspartame and its metabolites may directly influence cellular signaling pathways involved in cell growth, differentiation, and apoptosis, potentially contributing to carcinogenesis [14], with prenatal exposures showing potency in initiating carcinogenic processes [10]. These proposed mechanisms highlight the need for further research to clarify aspartame’s role in cancer development, particularly in light of the conflicting evidence from previous studies.

Despite these regulatory assurances, the debate over the safety of aspartame continues, underscoring the need for further research to clarify its potential health effects, particularly in relation to cancer [14]. This review aims to critically evaluate the existing literature on aspartame’s molecular mechanisms of action and its possible role as a carcinogen, providing insights that may inform future research and regulatory decisions regarding the use of this widely used artificial sweetener.

## 2. Methods

To provide a comprehensive and balanced analysis of aspartame’s potential carcinogenic and systemic effects, we conducted a literature review of peer-reviewed studies from a broad temporal range, with an emphasis on recent research published between 2018 and 2024 exclusively in English. However, foundational studies published prior to 2018 were also included when they offered essential mechanistic insights or historical context critical to understanding aspartame’s long-debated safety profile. Databases such as PubMed, ScienceDirect, and Google Scholar were used to identify relevant articles using keywords, including “aspartame,” “carcinogenicity,” “oxidative stress,” “formaldehyde,” and “artificial sweeteners.” Selected studies encompassed in vitro, in vivo, and epidemiological research that collectively addressed both systemic and molecular effects of aspartame. Articles were categorized thematically by mechanistic focus—such as lipid peroxidation, oxidative stress, immune modulation, formaldehyde toxicity, and gut microbiota alteration—to facilitate structured analysis.

## 3. Public Health Considerations of Aspartame

Aspartame is used globally in more than 6000 commercial food and beverage products, including diet sodas (e.g., Diet Coke and Diet Pepsi), sugar-free gums, tabletop sweeteners, and pharmaceutical formulations [7,14]. Its appeal stems from its intense sweetness—approximately 200 times that of sucrose—paired with negligible caloric content, making it a popular sugar substitute for individuals seeking weight management and glycemic control [3,14].

While aspartame’s low caloric value offers potential benefits in the context of rising obesity rates, its long-term safety remains controversial. Some randomized studies have shown that substituting sugar with non-nutritive sweeteners like aspartame may aid in modest weight loss and reduced calorie intake [15]. Others have argued that such sweeteners could paradoxically increase appetite or contribute to metabolic dysregulation [16,17]. Several observational and experimental studies have linked aspartame consumption with metabolic, cardiovascular, and gastrointestinal outcomes (Table 2).

From a metabolic standpoint, aspartame is commonly cited for its negligible effect on postprandial glucose and insulin levels, making it a common choice for diabetic diets [3,4]. However, several studies have questioned its metabolic safety. For example, in 2022, Debras et al. reported a significant association between high consumption of aspartame and increased risk of cardiovascular events in a cohort of over 100,000 French adults, particularly when intake exceeded typical dietary levels [18].

Moreover, observational data suggest a link between artificial sweeteners—including aspartame—and elevated risk of insulin resistance and non-alcoholic fatty liver disease (NAFLD), potentially mediated by gut microbiota changes and disruptions in incretin signaling [15,16]. Experimental models have also reported hepatotoxic effects and oxidative stress responses following aspartame ingestion [17,19].

Gastrointestinal symptoms such as bloating and nausea, often reported by consumers of artificially sweetened products, may be linked to these microbiota disturbances. While some clinical trials find these symptoms to be anecdotal or inconsistent, animal and in vitro studies increasingly support the plausibility of such mechanisms [15,19].

Despite ongoing debate, regulatory agencies such as the U.S. Food and Drug Administration (FDA) and the European Food Safety Authority (EFSA) maintain that aspartame is safe for human consumption when intake remains within the established Acceptable Daily Intake (ADI): 50 mg/kg body weight/day in the U.S. and 40 mg/kg in the EU [20,21]. According to the FDA, a 70 kg adult would need to consume more than 10–14 cans of diet soda daily to exceed the ADI [22].

Nevertheless, evidence of adverse effects occurring at or below these thresholds—particularly in vulnerable populations or under chronic exposure—has prompted renewed scrutiny by organizations such as the International Agency for Research on Cancer (IARC), which recently classified aspartame as “possibly carcinogenic to humans” (Group 2B) [22].

In conclusion, while aspartame remains widely used and approved by major regulatory agencies, concerns about its systemic effects—including metabolic, hepatic, cardiovascular, and gastrointestinal outcomes—warrant further investigation through well-controlled, longitudinal human studies.

**Table 2:** Summary of Studies and Regulatory Insights on the Public Health Impacts of Aspartame.

Study Type	Region	Mechanism of Action	Analyzed Risk	References
Review/General sources	Global	Low-calorie intake, sweet taste with negligible caloric content	Obesity management, a safe alternative to sugar	[4,7,14]
Carcinogenic classification by IARC	International (WHO)	Possible carcinogenic mechanisms, though evidence is limited	Cancer (Group 2B—possibly carcinogenic to humans)	[22]
Observational cohort study (NutriNet-Santé)	France	Association analysis using dietary records	Increased risk of cardiovascular and cerebrovascular diseases	[18]
Experimental and observational studies	Various (incl. US, Europe)	Disruption of cephalic response; modulation of insulin/incretin hormones; altered gut microbiota	Insulin resistance, NAFLD, hepatotoxicity, gastrointestinal issues	[15,16,17,19]

## 4. Controversies Surrounding Aspartame (Historical Concerns, Scientific Findings, and Regulations)

Public concerns and scientific scrutiny regarding aspartame’s safety have been shaped by historical debates, regulatory evaluations, and conflicting research findings. The introduction of artificial sweeteners began unexpectedly with the discovery of saccharin, a compound 300 times sweeter than sucrose, yet not metabolized by the human body [23]. Despite initial bans, saccharin was reinstated due to sugar shortages during World War I. The 1950s saw the emergence of cyclamate, another low-calorie sweetener, which was initially classified as GRAS (Generally Recognized as Safe) but was later banned after being linked to bladder cancer in rats [23]. Similarly, saccharin faced renewed scrutiny in the 1970s, leading to further regulatory actions [14]. Amid these developments, aspartame was discovered in 1965 by James M. Schlatter during research for an anti-ulcer drug. Although G.D. Searle & Co. sought approval in 1973, regulatory concerns delayed full approval until 1981 [6]. Early critics, including John Olney and James Turner, questioned aspartame’s safety, citing potential neurotoxic effects and risks for individuals with phenylketonuria [6,7]. Additionally, concerns about data inconsistencies in animal studies prompted further safety reviews, yet audits confirmed the validity of the studies conducted by Searle [20].

Further controversy arose from studies examining aspartame’s toxicity. In 1969, a study involving newborn monkeys was conducted, but the results were deemed inconclusive. A subsequent hamster study, intended to last 104 weeks, was halted at 46 weeks due to high mortality rates in both control and treatment groups, raising concerns from the Department of Health Education and Welfare (DHEW) [6]. A report by Jerome Bressler [24] highlighted data manipulation issues, including delayed corrections in glucose level discrepancies and the replacement of deceased test subjects. Searle argued these errors were accidental, and while Olney’s research into excitatory amino acid toxicity did not conclusively establish aspartame’s risks, it also did not confirm its safety [4,6]. These uncertainties have fueled ongoing debate, with researchers and the public remaining divided. The increasing availability of research and online discussions has further intensified scrutiny of aspartame’s potential carcinogenic effects, underscoring the need for continued scientific evaluation [6,20].

## 5. Aspartame’s Impact on Human Physiology and Pathways Implicated in Carcinogenesis

It is important to briefly mention the absorption and elimination of this substance in the human body. When consumed, aspartame is broken down by intestinal enzymes into various substances, mainly aspartic acid, phenylalanine, and methanol. These byproducts then go into the bloodstream and follow a certain metabolic pathway. For instance, methanol is processed in the liver, while aspartic acid and phenylalanine join the body’s pool of free amino acids. These metabolites are further used by tissues for different bodily functions and then excreted mainly through urine or converted to carbon dioxide and exhaled [25].

**Figure 1:**
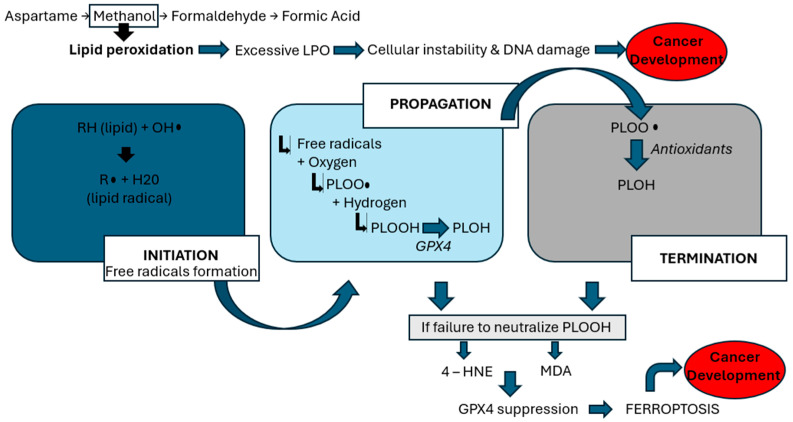
Lipid peroxidation, a mechanism through which aspartame induces cancer.

**Figure 2:**
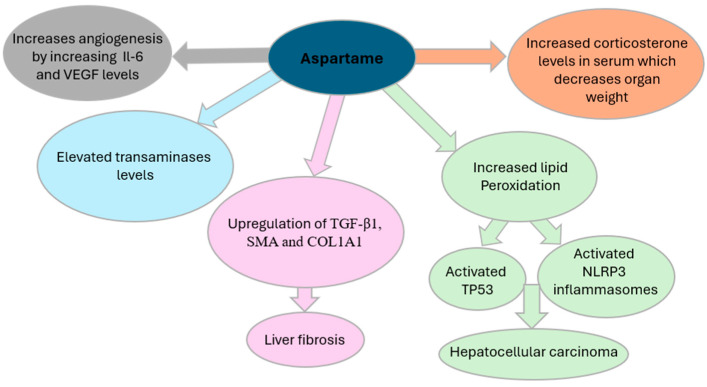
Aspartame carcinogenesis metabolic pathways.

The interplay of aspartame metabolites, oxidative stress, and genomic instability is proposed to contribute to carcinogenesis through multiple molecular pathways (Table 3). Recent research has explored aspartame’s interactions with various metabolic pathways, shedding light on its potential impact on human health. Notably, a study by Naik, AQ. et al. [17] highlights concerns regarding aspartame’s link to reproductive harm, emphasizing the role of methanol, a byproduct of aspartame metabolism. Methanol is metabolized into formaldehyde and formic acid, which generate reactive oxygen species (ROS)—highly reactive molecules that damage cellular structures. This oxidative environment initiates lipid peroxidation (LPO), a harmful chain reaction in which ROS attack polyunsaturated fats in cell membranes, leading to the formation of toxic lipid byproducts, loss of membrane integrity, and ultimately, cell death [8]. This process is a key mechanism by which aspartame may contribute to systemic toxicity and cancer risk. As demonstrated by Figure 1, the lipid peroxidation process encompasses three phases: initiation, propagation, and termination [17]. During the initiation phase, prooxidants, such as peroxyl radicals and hydroxyl radicals, generate lipid radicals. In the propagation phase, these radicals react with oxygen to form peroxy radicals (PLOO•), which interact with hydrogen atoms to produce phospholipid hydroperoxides (PLOOH). Glutathione peroxidase 4 (GPX4) typically converts PLOOH into phospholipid alcohol (PLOH) [16]. In the termination phase, antioxidants such as vitamin E neutralize PLOO•, yielding PLOH. However, when PLOOHs are not adequately counteracted by antioxidants or GPX4, they give rise to harmful byproducts such as 4-hydroxynonenal (4-HNE) and malondialdehyde (MDA) [17]. Moreover, this lipid peroxidation activates NOD-like-receptor-containing protein 3 (NLRP3) inflammasome and induces TP53, as described in Figure 2, both of which are pivotal in cancer development. NLRP3 activation has been strongly associated with HCC. Inhibiting NLRP3 has shown potential in slowing HCC cell proliferation [26]. In this setting, ferroptosis is a regulated form of cell death that depends on iron and is triggered by the buildup of lipid peroxides. It becomes a key final step in the chain of oxidative damage. When the cell’s antioxidant defense system, particularly when the enzyme GPX4, can no longer keep up, lipid peroxidation goes unchecked. This leads to the rupture of cell membranes and the release of pro-inflammatory signals. Ferroptosis is seen as the last cellular defense before cancer can develop, as it contributes to a tumor-friendly environment by promoting inflammation, DNA damage, and immune system evasion. Thus, excessive lipid peroxidation can trigger the process of oxidative stress, which refers to an imbalance between the production of ROS and the body’s antioxidant defenses [8], thus compromising cellular integrity and promoting inflammatory pathways that facilitate tumorigenesis—mechanisms that underlie aspartame’s potential role in cancer development.

A study conducted in 2023 by Griebsh et al. explored the potential neurotoxic effects of oxidative stress and DNA damage from aspartame and its metabolites (aspartic acid, phenylalanine, and methanol) on human SH-SY5Y neuroblastoma cells [19]. The researchers treated the cells with either aspartame or an equimolar mix of its three metabolites to simulate physiological digestion. Both treatments resulted in increased oxidative stress, as evidenced by elevated levels of ROS, upregulation of antioxidant genes (SOD1 and SOD2), and reduced cardiolipin levels—a key mitochondrial membrane lipid [19]. Mitochondrial damage was further confirmed by changes in morphology and increased expression of mitochondrial stress response genes like FIS1 and PINK1, indicating activation of mitophagy, a selective form of autophagy responsible for eliminating damaged mitochondria to preserve cellular homeostasis [19]. Additionally, the study found significant alterations in lipid metabolism [19]. Treated cells showed an accumulation of lipid droplets and a substantial rise in triacylglycerides and phospholipids, particularly phosphatidylcholines and phosphatidylethanolamines. While both aspartame and its metabolites triggered these effects, the complete aspartame molecule generally produced more pronounced changes [19]. These findings suggest that aspartame consumption may negatively impact neuronal lipid homeostasis and mitochondrial function, potentially contributing to the pathophysiology of neurodegenerative diseases. The authors recommend that the use of aspartame as a sugar substitute be reevaluated, especially concerning its possible impact on brain health [19].

In addition to that, oxidative stress, in general, directly influences the immune system’s ability to combat tumor progression by altering the function of adaptive and innate immune cells. Cytotoxic T lymphocytes (CD8^+^ T cells) play a key role in eliminating tumor cells through apoptosis, while CD4^+^ T cells regulate immune responses, with T helper subsets such as subsets 1 and 9 exerting antitumor effects, and regulatory T cells (Tregs) suppressing immune activity [27]. Additionally, natural killer (NK) cells contribute to tumor suppression. However, immune cell function is heavily dependent on metabolic balance, which is significantly disrupted by oxidative stress and lipid peroxidation byproducts, ultimately impairing antitumor immunity [27].

Lipid peroxidation products, particularly 4-HNE and MDA, further compromise immune responses by weakening T cell activation through disruption of the T cell receptor (TCR) signaling pathway. These byproducts induce ferroptosis, a form of non-apoptotic cell death driven by lipid peroxide accumulation, thereby suppressing cytokine production and diminishing cytotoxic T cell responses [27]. In CD8^+^ T cells, oxidized lipid uptake exacerbates peroxidation, activating the p38 kinase pathway, which is associated with T cell exhaustion and reduced antitumor activity [28]. Similarly, lipid peroxidation impairs NK cell function, weakening their ability to eliminate tumor cells [27]. In other words, aspartame and its byproducts may alter our immune system such that it would not be able to efficiently fight off tumors. This interplay between oxidative stress, lipid peroxidation, and immune dysfunction highlights the detrimental impact of metabolic imbalances in cancer progression.

In vivo studies have also investigated the impact of aspartame on immune responses. One study [29] identified aspartame as a potential contributor to angiogenesis, the physiological process of forming new blood vessels, a process critical for tumor progression. As highlighted in Figure 2, elevated concentrations of aspartame were associated with increased levels of inflammatory cytokines such as interleukin-6 (IL-6) and vascular endothelial growth factor (VEGF), along with their respective receptors. This promotes the formation of new blood vessels, supplying tumors with essential nutrients and facilitating their growth, invasion, and metastasis [21]. Aspartame consumption has been associated with increased serum corticosterone levels, contributing to oxidative stress and potential physiological disruptions. Elevated corticosterone has been linked to a reduction in organ weight [17] and a shorter disease-free interval in patients with breast and ovarian cancers [29]. In experimental models, glucocorticoids have been shown to suppress apoptosis in breast cancer cells, potentially facilitating tumor progression. Furthermore, exposure of ovarian cancer cells to cortisol has been observed to downregulate the tumor-suppressor genes ROBO1 and SLIT2 [29]. Notably, cortisol has also been reported to diminish the cytotoxic efficacy of chemotherapeutic agents such as paclitaxel, a primary treatment for ovarian cancer, thereby compromising therapeutic outcomes for cancer patients [29].

Another methanol metabolite, formaldehyde, has also been implicated in cytotoxicity [30]. Formaldehyde exposure has been shown to increase the number of cells undergoing apoptosis, which may contribute to the observed reduction in organ weight in animal studies [30]. Moreover, formaldehyde induces oxidative stress-mediated genetic alterations through its ability to interact with DNA [31]. Such changes could affect hematopoietic cells, potentially leading to malignancies such as leukemia.

On another note, aspartame has been shown to alter the expression of tumor-suppressor genes and oncogenes, form DNA crosslinks, and induce sister chromatid exchanges, suggesting potential carcinogenic effects at the molecular level. A study by Bayrak et al. investigated the impact of APM on heterochromatin regulation and stress response in the model organism Schizosaccharomyces pombe, focusing on the Swi6 protein, an ortholog of HP1, which plays a critical role in chromatin structure and gene silencing. Results demonstrated that aspartame downregulated Swi6 expression, affecting chromatin stability and stress response pathways. However, the magnitude of this effect was insufficient to conclusively classify aspartame as a strong carcinogenic agent. Additionally, metabolic assays showed that aspartame did not significantly alter glucose consumption or oxidative stress levels, suggesting a complex but limited role in cancer-related epigenetic modifications, which are heritable changes in gene expression that occur without alterations to the DNA sequence [32].

These findings suggest that aspartame may support the growth and progression of cancer by modulating cellular signaling pathways. Further research is warranted to elucidate the effects of aspartame on these pathways and its potential role in oncogenesis. Understanding these mechanisms could provide valuable insights into the relationship between aspartame and cancer, potentially paving the way for innovative strategies in cancer prevention and therapy.

DNA damage is caused by its metabolic breakdown into ROS [33]. Additionally, phenylalanine may influence tumor growth by promoting cell proliferation while inhibiting apoptosis [34]. However, the precise role of phenylalanine in cancer remains unclear, necessitating further research. Aspartame is also metabolized into methanol, which is further converted into formic acid [35]. A study by Czarnecka et al. examined this metabolic pathway, highlighting a correlation between aspartame exposure and reduced cell viability, as well as cytogenetic toxicity in red blood cells due to oxidative stress induced by formic acid [35]. However, conflicting reports have questioned this hypothesis, with some studies finding little to no association between aspartame consumption, oxidative stress, and genetic damage.

## 6. Carcinogenicity Studies and Methodologies

Experimental studies, including in vitro, in vivo, and epidemiological investigations, have played a crucial role in assessing the potential carcinogenicity of aspartame. While some research has suggested a correlation between aspartame intake and tumor development, conflicting findings have led to ongoing scientific debate.

Early animal studies, such as those conducted by Searle, suggested a potential involvement of aspartame consumption with cancer. For instance, one study reported an increased incidence of tumors, particularly lymphomas and leukemias, in rats exposed to aspartame [36,37]. Similarly, research by the European Ramazzini Foundation found higher occurrences of these malignancies in rats exposed to aspartame from embryonic stages until death [38]. However, other studies, such as a comprehensive review by the EFSA in 2013, have contradicted these findings, having found no conclusive evidence linking aspartame to cancer [39]. Likewise, the National Toxicology Program in 2005 did not establish any association between aspartame intake and tumor development in rats [9].

Concerns regarding aspartame’s potential carcinogenicity largely stem from its metabolism into formaldehyde, a compound known to be carcinogenic. This hypothesis suggests that chronic exposure to formaldehyde may contribute to cancer initiation and progression [31]. Furthermore, studies have proposed that aspartame may promote the growth of pre-existing tumors [37] and facilitate crosslinking of proteins and nucleic acids, potentially compromising cellular integrity [40]. Since formaldehyde is a natural byproduct of aspartame metabolism through oxidative demethylation [41], its role in DNA damage and carcinogenesis warrants further investigation [36]. However, definitive validation of this hypothesis remains elusive and requires more rigorous research.

Another metabolite of aspartame, phenylalanine, has also been investigated for its possible role in carcinogenesis [26]. As a naturally occurring amino acid, phenylalanine has been linked to cancer development through mechanisms such as oxidative stress and DNA damage caused by its metabolic breakdown into ROS [33]. Additionally, phenylalanine may influence tumor growth by promoting cell proliferation while inhibiting apoptosis [39]. However, the precise role of phenylalanine in cancer remains unclear, necessitating further research. Aspartame is also metabolized into methanol, which is further converted into formic acid [9]. A study by Czarnecka et al. examined this metabolic pathway, highlighting a correlation between aspartame exposure and reduced cell viability, as well as cytogenetic toxicity in red blood cells due to oxidative stress induced by formic acid [9]. However, conflicting reports have questioned this hypothesis, with some studies finding little to no association between aspartame consumption, oxidative stress, and genetic damage.

The impact of aspartame on mammalian cells has also been evaluated. Research investigating chromosomal abnormalities following aspartame exposure demonstrated a dose-dependent increase in aberration frequency, with a fourfold rise at the highest administered dose [40]. However, studies on specific-pathogen-free male mice exposed to aspartame doses between 500 and 2000 mg/kg found no increase in micronucleated polychromatic erythrocytes in bone marrow, suggesting no mutagenic effects [38]. In contrast, some in vivo studies have reported opposing results, such as an increased presence of micronucleated erythrocytes in Swiss albino mice and chromosomal abnormalities in bone marrow [36].

Research conducted by the Cancer Research Center of the Ramazzini Institute linked aspartame consumption to malignancies in multiple rat organs, demonstrating a dose–response relationship in which cancer risk increased at concentrations higher than typical dietary exposure [36,38]. Soffritti et al. [36] further observed heightened sensitivity to aspartame in rodents exposed prenatally, suggesting long-term risks. Landrigan et al. [10] suggested that this dose–response relationship could be relevant to humans, even at moderate intake levels close to the acceptable daily intake (ADI) of 40–50 mg/kg/day. Conversely, Magnuson et al. [42] argued that human aspartame consumption remains well below the ADI and is unlikely to pose a carcinogenic risk. Their study demonstrated that even high doses of aspartame did not elevate postprandial phenylalanine and aspartic acid levels beyond normal physiological ranges. It is essential that all the above-mentioned studies be validated in human models.

Epidemiological studies have presented mixed results regarding aspartame intake and cancer risk. Some research has indicated higher cancer rates among individuals who consume substantial amounts of aspartame [18]. In vitro studies suggest that high doses of aspartame can exhibit cytotoxic effects on human colorectal carcinoma cells and promote angiogenesis, potentially contributing to tumor progression [43]. Some epidemiological studies have linked aspartame intake to non-Hodgkin lymphomas and multiple myelomas in men [18]. A cohort study performed by Debras et al. in 2022 aimed to investigate the association between aspartame intake and cancer risk [23]. Drawing on data from 102,865 French adults over a median follow-up of 7.8 years, the researchers assessed dietary intake of artificial sweeteners—including aspartame, acesulfame-K, and sucralose—using repeated 24 h food records linked to product composition databases [23]. They found that higher intake of total artificial sweeteners was associated with an increased overall cancer risk (hazard ratio [HR] = 1.13), with specific associations for aspartame (HR = 1.15) and acesulfame-K (HR = 1.13). Aspartame was also linked to elevated risks of breast cancer (HR = 1.22) and obesity-related cancers (HR = 1.15) [23]. The study’s strengths include its large sample size, detailed dietary data, and robust statistical adjustments for confounding variables. However, it notes limitations such as potential selection bias, limited generalizability to the broader population, and the observational nature of the data, which precludes definitive causal conclusions [10]. The findings suggest artificial sweeteners, particularly aspartame and acesulfame-K, may be modifiable risk factors for cancer and warrant further investigation by health authorities [23].

Conversely, research by Guercio et al. [44] found no increased cancer risk and even reported a reduced risk of cancer recurrence in individuals with stage III colon cancer who consumed artificially sweetened beverages.

Despite these findings, the IARC has classified the evidence linking aspartame consumption to cancer as insufficient, with the exception of HCC [45]. Consequently, the EFSA has questioned the significance of observed chromosomal abnormalities in certain studies, suggesting that they may be secondary cytotoxic effects rather than direct DNA damage [39]. The EFSA also highlighted methodological concerns in study designs, particularly in assessing cytotoxicity and treatment timing. As a result, current in vivo data fail to provide definitive evidence of aspartame’s oncogenic potential.

In conclusion, both in vitro and in vivo studies have explored the genotoxic and carcinogenic effects of aspartame, yet findings remain inconclusive. While some research suggests potential risks, epidemiological evidence has not established a definitive link between aspartame consumption and cancer risk. Based on the current scientific literature, aspartame cannot be definitively classified as genotoxic or carcinogenic. Given the inconsistencies in the available data, further rigorous investigations are needed to clarify aspartame’s potential role in carcinogenesis.

## 7. Limitations and Future Perspectives

The interpretation of existing findings is constrained by considerable variability in study design, including differences in model systems (animal vs. human), dosage levels, duration of exposure, and methodological rigor. Additionally, translating high-dose animal data to typical human consumption remains a challenge. These limitations emphasize the need for well-controlled, long-term human studies to more accurately assess the health risks associated with aspartame.

To address the current controversy surrounding aspartame’s safety profile and potential carcinogenic effects, future research should prioritize large-scale, longitudinal human studies with robust dietary tracking and biomarker analysis. Integrating technologies such as genomics, metabolomics, and microbiomics could provide deeper mechanistic insights into how aspartame interacts with human metabolism, immune function, and gene expression. Additionally, standardized experimental protocols and harmonized exposure metrics across studies are essential to improve reproducibility and comparability. Collaborative efforts between regulatory agencies, academic institutions, and independent research bodies will be key to resolving uncertainties and guiding evidence-based public health recommendations.

## 8. Conclusions

In summary, this mini-review has examined the potential link between aspartame and carcinogenesis. While some studies suggest a possible association, the overall body of evidence remains inconclusive. The mechanisms explored—ranging from oxidative stress and lipid peroxidation to immune suppression and epigenetic modulation—highlight the complexity of aspartame’s interaction with human physiology. Regulatory agencies maintain that aspartame is safe within established intake limits, yet ongoing scientific debate underscores the importance of further investigation.

## Figures and Tables

**Table 1:** Major metabolic derivatives of aspartame, along with their biological function and relevance.

Derivative	Chemical Formula	Chemical Structure	Function/Relevance
Aspartic Acid	C_4_H_7_NO_4_	HO | HOOC-CH_2_-CH-COOH | NH_2_	Excitatory neurotransmitter; implicated in neurotoxicity when in excess [5]
Phenylalanine	C_9_H_11_NO_2_	NH_2_ | HOOC-CH-CH_2_- | 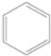	Essential amino acid; precursor of dopamine and norepinephrine; neurotoxic in PKU [6,7]
Methanol	CH_3_OH	H | H-C-OH | H	Metabolized to formaldehyde and formic acid; toxic at high doses [8]
Formaldehyde	CH_2_O	H | C=O | H	DNA crosslinking agent; classified as a Group 1 carcinogen by IARC [6]
Formic Acid	HCOOH	O ║ H-C-OH	Inhibits mitochondrial cytochrome oxidase; associated with metabolic acidosis [9]

**Table 3:** Summary table of studies performed to assess aspartame’s effect on molecular pathways leading to carcinogenesis.

Study Type	Substance/Metabolite	Evaluated Mechanism	Bioassay Model	Related Cancer Stage	References
In vitro (cell culture)	Aspartame and Metabolites (aspartic acid, phenylalanine, methanol)	Oxidative stress, mitochondrial damage, lipid metabolism alterations	Human SH-SY5Y neuroblastoma cells	Early tumorigenesis (cellular stress)	[19]
Review	Oxidative stress byproducts (4-HNE, MDA)	Immune dysfunction (TCR signaling disruption, ferroptosis, T/NK cell exhaustion)	N/A (Theoretical)	Tumor immune evasion	[8,27,28]
In vivo (animal/clinica)	Aspartame	Angiogenesis (IL-6/VEGF upregulation), corticosterone-induced apoptosis suppression	Rodent models and human cancer patients	Tumor progression/metastasis	[17,21,29]
In vitro (cell culture)	Formaldehyde (methanol metabolite)	DNA damage, oxidative stress, and apoptosis	Hematopoietic cells	Leukemogenesis	[30,31]
In vitro (yeast model)	Aspartame	Epigenetic dysregulation (Swi6/HP1 downregulation)	Schizosaccharomyces pombe yeast	Chromatin instability	[32]

## Abbreviations

The following abbreviations are used in this manuscript:

ADI	Acceptable Daily Intake
APM	Aspartame
CD8^+^ T cells	Cytotoxic T Lymphocytes
CD4^+^ T cells	Helper T Lymphocytes
FDA	Food and Drug Administration
EFSA	European Food Safety Authority
GPX4	Glutathione Peroxidase 4
HCC	Hepatocellular Carcinoma
HP1	Heterochromatin Protein 1
IL-6	Interleukin-6
IARC	International Agency for Research on Cancer
LPO	Lipid Peroxidation
MDA	Malondialdehyde
NAFLD	Nonalcoholic Fatty Liver Disease
NLRP3	NOD-like Receptor Pyrin Domain Containing 3
NK cells	Natural Killer Cells
PINK1	PTEN-induced Kinase 1
PLOOH	Phospholipid Hydroperoxide
PLOO•	Phospholipid Peroxyl Radical
PLOH	Phospholipid Alcohol
ROS	Reactive Oxygen Species
SOD1/SOD2	Superoxide Dismutase 1/2
TCR	T Cell Receptor

## References

[1] Gloria M.B.A., Caballero B. (2003). Aspartame. Encyclopedia of Food Sciences and Nutrition.

[2] Ellwein L.B., Cohen S.M. (2008). The Health Risks of Saccharin Revisited. Crit. Rev. Toxicol..

[3] Choudhary A.K., Pretorius E. (2017). Revisiting the Safety of Aspartame. Nutr. Rev..

[4] Guy R.C., Wexler P. (2014). Aspartame. Encyclopedia of Toxicology.

[5] Choi D.W., Viseskul V., Amirthanayagam M., Monyer H. (1989). Aspartate Neurotoxicity on Cultured Cortical Neurons. J. Neurosci. Res..

[6] Nill A. (2000). The History of Aspartame.

[7] Shaher S.A.A., Mihailescu D.F., Amuzescu B. (2023). Aspartame Safety as a Food Sweetener and Related Health Hazards. Nutrients.

[8] Nam T.-G. (2011). Lipid Peroxidation and Its Toxicological Implications. Toxicol. Res..

[9] National Toxicology Program (NTP) (2005). NTP Report on the Toxicology Studies of Aspartame in Genetically Modified Mice.

[10] Landrigan P.J., Straif K. (2021). Aspartame and Cancer—New Evidence for Causation. Environ. Health.

[11] Bayrak N., Yilmazer M., Palabiyik B. (2025). Aspartame Safety and Health Implications: A Comprehensive Review. Int. Food Res. J..

[12] Knowledge Based Value (KBV) Research (2023). Global Aspartame Market Size, Share & Industry Trends Analysis Report (2023–2029).

[13] Hou X., Chen D. (2024). Aspartame Carcinogenic Potential Revealed Through Network Toxicology and Molecular Docking Insights. Sci. Rep..

[14] Malkan S. (2025). Aspartame: Decades of Science Point to Serious Health Risks. Environmental Health News.

[15] Liauchonak I., Qorri B., Dawoud F., Riat Y., Szewczuk M.R. (2019). Non-Nutritive Sweeteners and Their Implications on the Development of Metabolic Syndrome. Nutrients.

[16] Kossiva L., Karaglani K., Christodouli F., Soldatou A., Karanasios S., Karavanaki K. (2024). Chronic Use of Artificial Sweeteners: Pros and Cons. Nutrients.

[17] Naik A.Q., Zafar T., Shrivastava V.K. (2023). The Impact of Non-Caloric Artificial Sweetener Aspartame on Female Reproductive System in Mice Model. Reprod. Biol. Endocrinol..

[18] Debras C., Chazelas E., Srour B., Druesne-Pecollo N., Esseddik Y., de Edelenyi F.S., Agaësse C., De Sa A., Lutchia R., Gigandet S. (2022). Artificial Sweeteners and Cancer Risk: Results from the NutriNet-Santé Population-Based Cohort Study. PLoS Med..

[19] Griebsch L.V., Theiss E.L., Janitschke D., Erhardt V.K.J., Erhardt T., Haas E.C., Kuppler K.N., Radermacher J., Walzer O., Lauer A.A. (2023). Aspartame and Its Metabolites Cause Oxidative Stress and Mitochondrial and Lipid Alterations in SH-SY5Y Cells. Nutrients.

[20] Butchko H.H., Kotsonis F.N. (1991). Acceptable Daily Intake vs Actual Intake: The Aspartame Example. J. Am. Coll. Nutr..

[21] Pati S., Irfan W., Jameel A., Ahmed S., Shahid R.K. (2023). Obesity and Cancer: A Current Overview of Epidemiology, Pathogenesis, Outcomes, and Management. Cancers.

[22] Stiepan D. Mayo Clinic Expert Weighs in on WHO Labeling Aspartame Sweetener as Low, but Possible, Cancer Risk. *Mayo Clinic News Network*, 14 July 2023. https://newsnetwork.mayoclinic.org/discussion/mayo-clinic-expert-weighs-in-on-who-labeling-aspartame-sweetener-as-low-but-possible-cancer-risk/.

[23] Debras C., Chazelas E., Sellem L., Porcher R., Druesne-Pecollo N., Esseddik Y., de Edelenyi F.S., Agaësse C., De Sa A., Lutchia R. (2022). Artificial Sweeteners and Risk of Cardiovascular Diseases: Results from the Prospective NutriNet-Santé Cohort. BMJ.

[24] Bressler J. Histroy of the U.S. Food and Drug Administration. https://www.fda.gov/media/80995/download/.

[25] Pang M.D., Goossens G.H., Blaak E.E. (2021). The Impact of Artificial Sweeteners on Body Weight Control and Glucose Homeostasis. Front. Nutr..

[26] International Agency for Research on Cancer (IARC) (2023). Carcinogenicity of Aspartame, Methyleugenol, and Isoeugenol.

[27] Xiao L., Xian M., Zhang C., Guo Q., Yi Q. (2023). Lipid Peroxidation of Immune Cells in Cancer. Front. Immunol..

[28] Medina K.L. (2016). Overview of the Immune System. Handb. Clin. Neurol..

[29] Schrepf A., Thaker P.H., Goodheart M.J., Bender D., Slavich G.M., Dahmoush L., Penedo F., DeGeest K., Mendez L., Lubaroff D.M. (2015). Diurnal Cortisol and Survival in Epithelial Ovarian Cancer. Psychoneuroendocrinology.

[30] Nakao H., Umebayashi C., Nakata M., Nishizaki Y., Noda K., Okano Y., Oyama Y. (2003). Formaldehyde-Induced Shrinkage of Rat Thymocytes. J. Pharmacol. Sci..

[31] Kawanishi M., Matsuda T., Yagi T. (2014). Genotoxicity of Formaldehyde: Molecular Basis of DNA Damage and Mutation. Front. Environ. Sci..

[32] Bayrak B., Yilmazer M., Palabiyik B. (2020). Artificial Food Sweetener Aspartame Induces Stress Response in Model Organism *Schizosaccharomyces pombe*. Toxicol. Rep..

[33] Saleh E.A.M., Al-Dolaimy F., Almajidi Y.Q., Baymakov S., Kader M.A., Ullah M.I., Abbas A.H.R., Khlewee I.H., Bisht Y.S., Alsaalamy A.H. (2023). Oxidative Stress Affects the Beginning of the Growth of Cancer Cells Through a Variety of Routes. Pathol. Res. Pract..

[34] (2022). Novel L-Phenylalanine Dipeptide Inhibits Prostate Cancer Growth via Targeting DUSP1 and TNFSF9. Int. J. Mol. Sci..

[35] Czarnecka K., Pilarz A., Rogut A., Maj P., Szymańska J., Olejnik Ł., Szymański P. (2021). Aspartame—True or False? Narrative Review of Safety Analysis of General Use in Products. Nutrients.

[36] Soffritti M., Belpoggi F., Degli Esposti D., Lambertini L., Tibaldi E., Rigano A. (2006). First Experimental Demonstration of the Multipotential Carcinogenic Effects of Aspartame Administered in the Feed to Sprague-Dawley Rats. Environ. Health Perspect..

[37] Soffritti M., Belpoggi F., Degli Esposti D., Lambertini L. (2006). Results of a Long-Term Carcinogenicity Bioassay on Sprague-Dawley Rats Exposed to Sodium Arsenite Administered in Drinking Water. Ann. N. Y. Acad. Sci..

[38] Soffritti M., Belpoggi F., Tibaldi E., Esposti D.D., Lauriola M. (2007). Life-Span Exposure to Low Doses of Aspartame Beginning During Prenatal Life Increases Cancer Effects in Rats. Environ. Health Perspect..

[39] EFSA Panel on Food Additives and Nutrient Sources Added to Food (ANS) (2013). Scientific Opinion on the Re-Evaluation of Aspartame (E 951) as a Food Additive. EFSA J..

[40] McGhee J.D., von Hippel P.H. (1977). Formaldehyde as a Probe of DNA Structure. 3. Equilibrium Denaturation of DNA and Synthetic Polynucleotides. Biochemistry.

[41] Pontel L.B., Rosado I.V., Burgos-Barragan G., Garaycoechea J.I., Yu R., Arends M.J., Chandrasekaran G., Broecker V., Wei W., Liu L. (2015). Endogenous Formaldehyde Is a Hematopoietic Stem Cell Genotoxin and Metabolic Carcinogen. Mol. Cell.

[42] Magnuson B.A., Burdock G.A., Doull J., Kroes R.M., Marsh G.M., Pariza M.W., Spencer P.S., Waddell W.J., Walker R., Williams G.M. (2007). Aspartame: A Safety Evaluation Based on Current Use Levels, Regulations, and Toxicological and Epidemiological Studies. Crit. Rev. Toxicol..

[43] Alleva R., Borghi B., Santarelli L., Strafella E., Carbonari D., Bracci M., Tomasetti M. (2011). In Vitro Effect of Aspartame in Angiogenesis Induction. Toxicol. Vitro.

[44] Guercio B.J., Zhang S., Niedzwiecki D., Li Y., Babic F.A., Morales-Oyarvide V., Saltz L.B., Mayer R.J., Mowat R.B., Whittom R. (2018). Associations of Artificially Sweetened Beverage Intake with Disease Recurrence and Mortality in Stage III Colon Cancer: Results from CALGB 89803 (Alliance). PLoS ONE.

[45] Stepien M., Duarte-Salles T., Fedirko V., Trichopoulou A., Lagiou P., Bamia C., Overvad K., Tjønneland A., Hansen L., Boutron-Ruault M.-C. (2016). Consumption of Soft Drinks and Juices and Risk of Liver and Biliary Tract Cancers in a European Cohort. Eur. J. Nutr..

